# Supplementary material for: Intraoperative microseizure detection using a high-density micro-electrocorticography electrode array
Source: Brain Commun. 2022 May 27;4(3):fcac122. doi: 10.1093/braincomms/fcac122 (PMC9155612; doi:10.1093/braincomms/fcac122)
Supplement: fcac122_Supplementary_Data [file fcac122_supplementary_data.pdf]

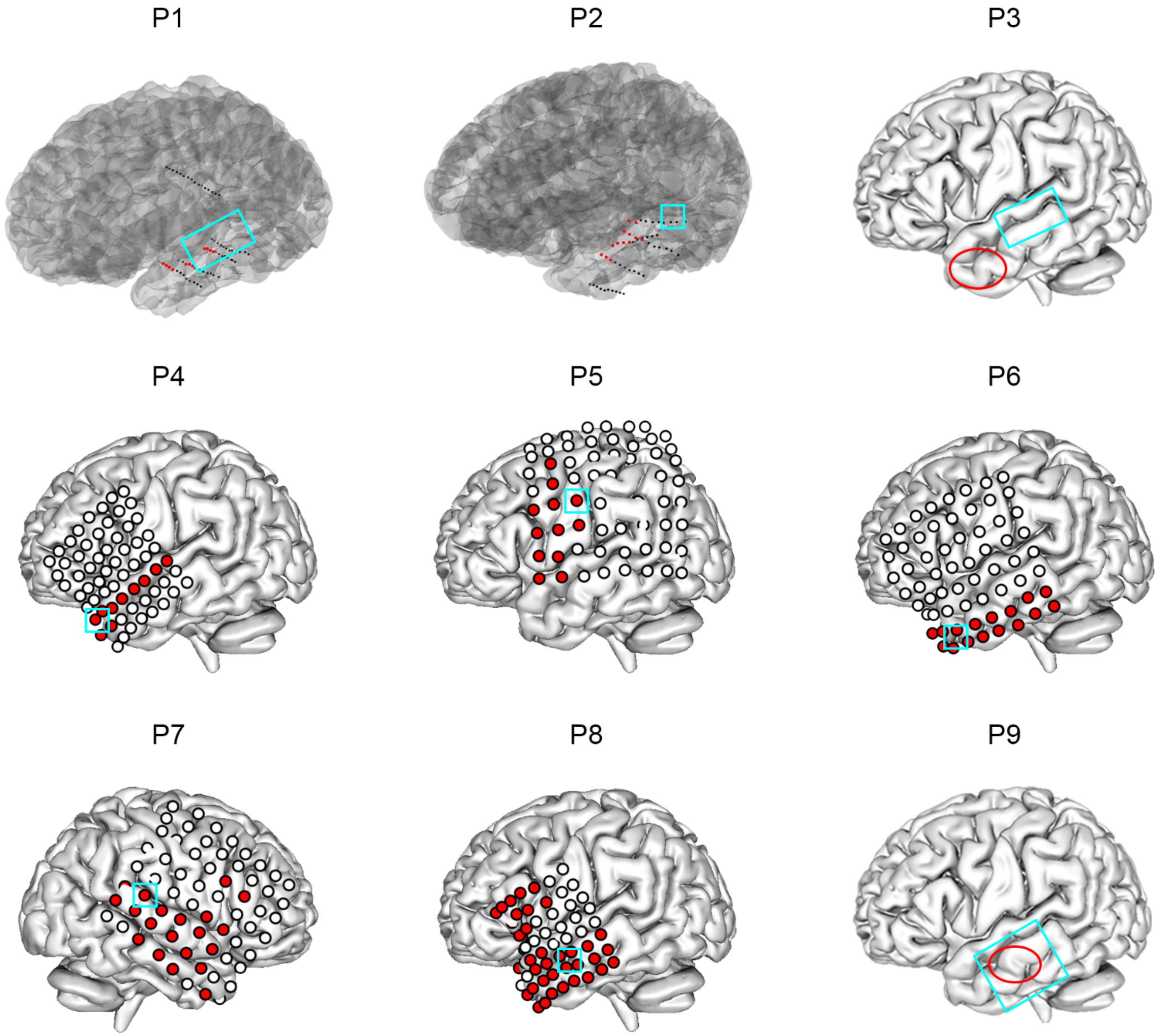

Supplementary Figure 1. Locations of intraoperative µECoG recordings in epileptic subjects relative to clinically identified epileptic tissue. For each epileptic subject (P1-P9), red markings indicate clinically identified areas of seizure onset or early spread (5-15 sec post seizure onset) determined during preoperative monitoring or a lesion identified in the MRI. Blue markings indicate the location of each µECoG array during intraoperative recordings. P1 and P2 were clinically monitored with sEEG electrodes showing mesial epileptic onset and spread, P3 and P9 had lesions identified in the MRI, and P4-P8 were monitored with clinical standard ECoG (grid) arrays.

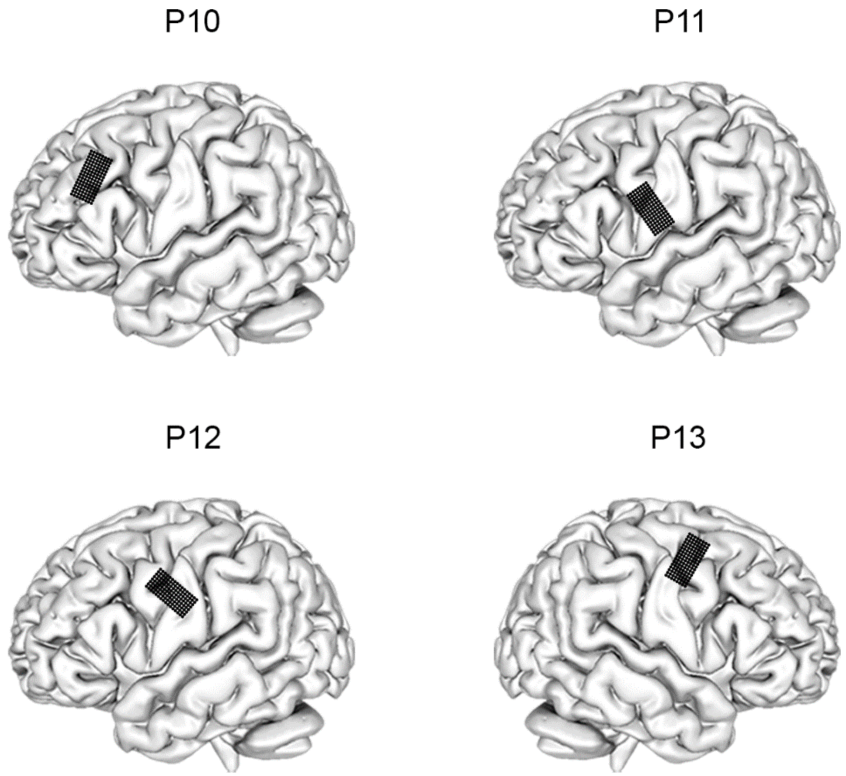

Supplementary Figure 2. Locations of intraoperative µECoG recordings in control subjects. For each non-epileptic control subject (P10-P13), a rectangular grid indicates the location of the µECoG array placed through a burr hole onto the cortical surface during DBS implantation surgery for treatment of a movement disorder.

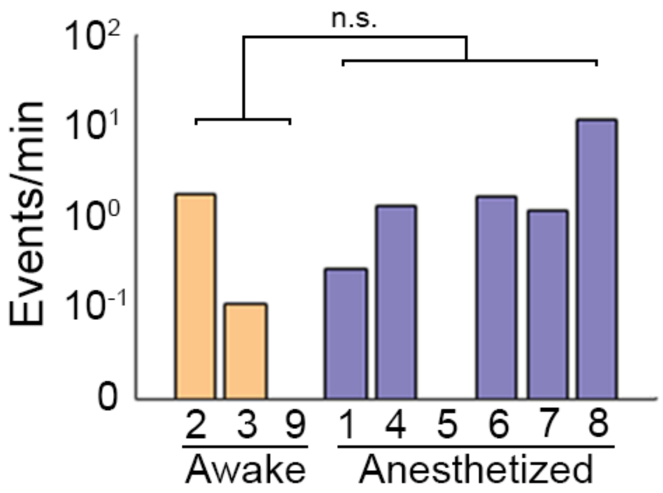

Supplementary Figure 3. Microseizure rates do not differ significantly between awake (*n*=3) and anesthetized (*n*=6) epilepsy patients. Microseizure rates in awake (P2, P3, P9) and anesthetized (P1, P4, P5, P6, P7, P8) epilepsy patients were compared. Rates did not differ in mean (permutation test, test statistic = 2.0483, P=0.5703), rank (Mann-Whitney U test, U_1=12.5, U_2=5.5, P=0.6190), or distribution (Kolmogorov-Smirnov test, D^*=0.5, P=0.5344). n.s. = not significant.

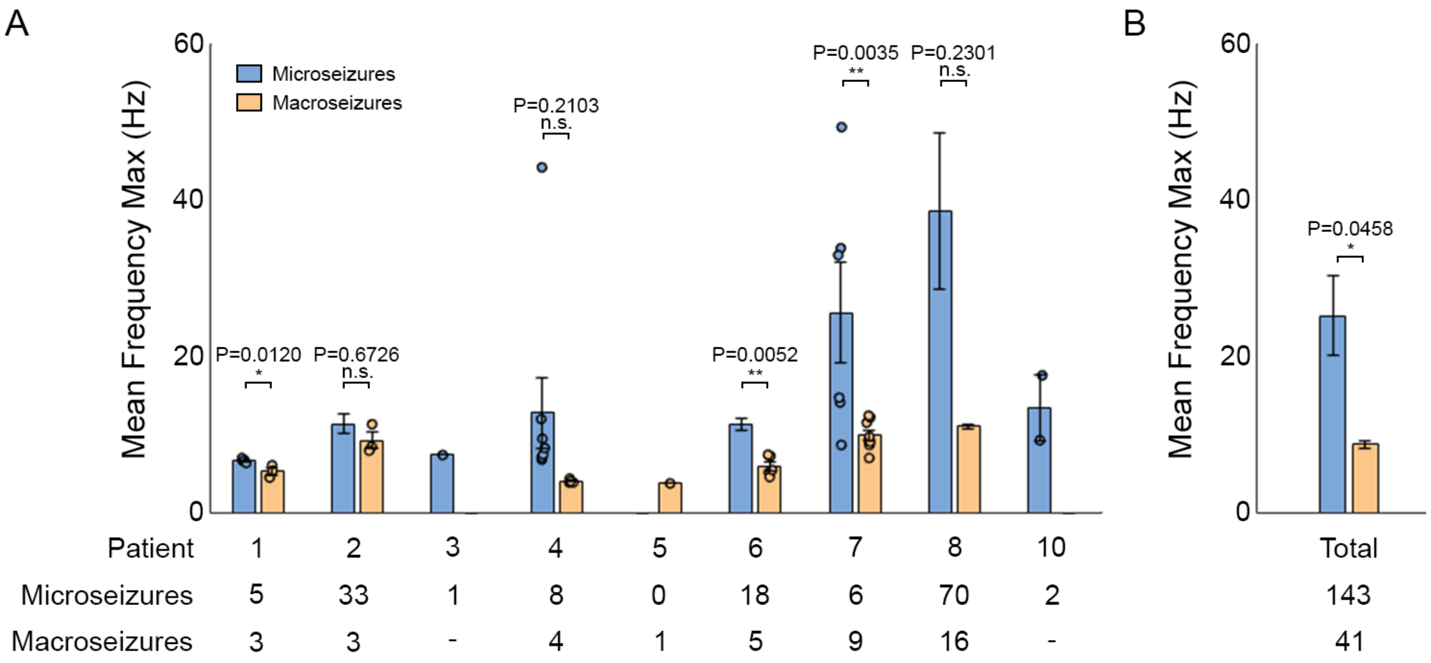

Supplementary Figure 4. Microseizures have higher mean Frequency Maxes than macroseizures. A) The mean Frequency Max of microseizures was compared to the mean Frequency Max of clinical macroseizures for each patient using a two-sided permutation test (P<0.05, n.s. = not significant). The number of microseizures and macroseizures is listed below each patient number. For P3, no macroseizure recordings were available since the patient did not undergo intracranial electrographic monitoring. In P5 and P9, no microseizures were identified. P10 was a non-epileptic control and therefore no macroseizure data was available. B) Comparison between the mean Frequency Max across all microseizure events (143) and clinical macroseizures (41). The mean Frequency Max of microseizures (25.3 ± 5.0 Hz) was significantly higher than the mean Frequency Max of macroseizures (8.9 ± 0.5 Hz) by a two-sided permutation test (P = 0.0458)
